# A practical fully automated radiosynthesis of [^18^F]Flurpiridaz on the module modular lab-pharmtracer without external purification

**DOI:** 10.1186/s41181-022-00182-z

**Published:** 2022-11-05

**Authors:** Kurtulus Eryilmaz, Benan Kilbas

**Affiliations:** Moltek A. S. Gebze Organize Sanayi, 41400 Gebze, Kocaeli Turkey

**Keywords:** Automated synthesis, Cardiac PET, ^18^F, [^18^F]Flurpiridaz

## Abstract

**Background:**

[^18^F]Flurpiridaz is a promising novel cardiac PET imaging tracer formed by the radiolabeling of pyridaben derivative with fluorine-18. Clinical studies on [^18^F]Flurpiridaz are currently at the phase III level for the assessment of MPI. Providing high image quality thanks to its relatively long half-life, F-18 is a high-potential radionuclide for the early detection of CAD. In this study, we aimed to develop a fully automated synthesis of [^18^F]Flurpiridaz without further preparative HPLC purification.

**Results:**

Precursor **6** was obtained by multi-step synthesis starting from mucochloric acid (**1**) as a sole product with 35% yield and identified by spectroscopic measurement. Manually cold labeling experiments were performed using the stable isotope [^19^F]F, and TBA-HCO_3_ PTC provided desirable fluorinated compound with high yield. A fully automated [^18^F]Flurpiridaz synthesis on the ML-PT device provided 55–65% radiochemical yield with more than 98% radiochemical purity. The final product purification method demonstrated that [^18^F]Flurpiridaz could be obtained without an external preparative HPLC system as a pharmaceutical quality.

**Conclusion:**

A novel and fascinating strategy was developed for the fully automated synthesis of [^18^F]Flurpiridaz (**7**) on ML PT. Organic synthesis of precursor **6** was achieved with a desirable yield and characterized by NMR and HR-MS. A detailed set of cold experiments were completed for optimization conditions before hot trials and TBA-HCO_3_ increased molar activity with a minimum amount of side products. Radiolabeling showed that our self-designed automated synthesis method enables high radiochemical yield and radiochemical purity for the production of [^18^F]Flurpiridaz. The desirable radiopharmaceutical quality of the product was obtained without using an additional preparative HPLC system. [^18^F]Flurpiridaz (**7**) preserved its stability within 12 h and final specifications were consistent with the acceptance criteria in Ph. Eur. regulations.

## Background

**Scheme 1:**
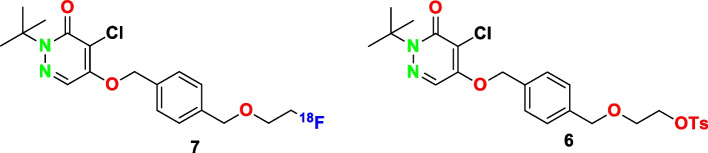
Molecular Structures of [^18^F]Flurpiridaz **(7)** and its precursor **6**

Coronary artery disease (CAD) has been frequently observed worldwide which causes mortality due to the coronary artery atherosclerosis (Fowkes et al. 2017). Increase the number of CAD led to investigations of different treatment approaches (Davidson et al. 2018). Myocardial perfusion imaging (MPI) tools, such as cardiac magnetic resonance (CMR), single-photon emission computed tomography (SPECT) and positron emission tomography (PET) have for the past decades attracted increasing interest for an accurate diagnosis of coronary heart disease (Danad et al. 2017). [^99^Tc]Tc-Sestamibi, approved agent by FDA is well known SPECT-tracer as a diagnostic agent for clinics. Even though it has been widely used as a diagnostic agent, it has some limitations such as less spatial resolution and additional correction measurement (Wu et al. 2009). Recently, positron emission tomography (PET) has been most widely utilized in MPI due to the better diagnostic performance, high resolution of images and lower radiation exposure (Di Carli et al. 2007). Even though, [^82^Rb]Rb (Huang et al. 1989), [^13^N]N (Schelbert et al. 1981), [^11^C]C (Croteau et al. 2012), and [^15^O]O (Bergmann et al. 1989) have been exhibited as PET radionuclides for the detection of MPI, shorter half-life and higher cost cause a limit of their widespread use. For example, [^82^Rb]Rb generator is replaced every one month with a high amount of cost (€ 30.000 per one month). Recently, [^68^Ga]Ga-DOTA has been exhibited as an alternative PET agent for MPI in small animals however comprehensive clinical trials are necessary to be a reliable diagnostic agent (Autio et al. 2020). Those limitations of available PET tracers have led to a need for an optimal perfusion agent with improved properties. [^18^F]Flurpiridaz is an ideal pyridaben-derivative ^18^F-labeled MPI agent which binds to mitochondrial complex (MC) I inhibitor with high affinity (Scheme 1) (Maddahi et al. 2020). Preclinical and clinical trials have indicated that [^18^F]Flurpiridaz is clinically safe and enhances a high-resolution quality of image and particularly it is more favorable in women, obese patients and patients with multi-vessel disease. (Berman et al. 2013) Besides, one cyclotron site can be sufficient for dose distribution to many different nuclear medicine centers due to the relatively long physical half-life feature of Fluorine-18.

To the best our knowledge, detailed radiolabeling study of [^18^F]Flurpiridaz **(7)** has not been published yet despite expected popularity of its use in many clinics. In this article, the scope was to demonstrate a fully automated synthesis of [^18^F]Flurpiridaz **(7)** radiopharmaceutical considering radiation safety and in accordance with regulations of radiopharmaceutical preparations by using commercially available an automated synthesis platform namely Modular Lab-PharmTracer (ML PT) for the first time.

## Methods

### Materials

Chemicals and solvents were supplied from Sigma-Aldrich (Germany) and directly used without any further purification. Reactions were magnetically stirred and monitoring was determined by TLC performed on Merck TLC plates (silica gel 60 F254). The resulting TLC spots were detected with UV light (λ = 254 nm and 360 nm). The ^1^H NMR spectra were recorded on a Bruker BioSpin (DPX-400) instrument and mass spectra were recorded on an Agilent 6430 LC-MS/MS spectrometer equipped with an electrospray ionization source (ESI). HPLC analyses were monitored by combined Shimadzu LC20A and Eckert & Ziegler HPLC Scan devices. Modular Lab-Pharm-Tracer synthesis device (Eckert & Ziegler Eurotope, Berlin, Germany) was used for labeling synthesis.

### Chemistry

#### Synthesis of precursor of [^18^F]Flurpiridaz (7) [2-(4-((1-tert-Butyl-5-chloro-6-oxo-1,6-dihydropyridazine-4-yloxy)methyl)benzyloxy)ethyl-4- methylbenzensulfonate] (6)

Precursor **6** was synthesized according to the literature procedures with few changes (Purohit et al. 2008; Nagel 2014) Briefly, to a mixture of mucochloric acid (**1**) (1.18 g, 6.98 mmol) and Na_2_CO_3_ (0.33 g, 3.11 mmol) in 15 ml of distilled water was added *tert*-butylhydrazine hydrochloride (0.86 g, 6.90 mmol) in ice-water bath and reaction mixture was stirred for about 4 h. White precipitate was washed by water and dried under reduced vacuum after filtration. Then, 13.2 ml of benzene and acetic acid (1.86 g, 30,95 mmol) were added and reaction was kept at 40 °C for 4 h. Organic phase was extracted with 10 ml of water and washed by 5 ml of 1.25 M NaOH(aq), 5 ml of 5 M HCl(aq) and 10 ml of water respectively. 0.83 g of DCP (**2**) was obtained as an orange solid. 1.0 g of DCP (**2**) (4,53 mmol) was dissolved in 15 ml of dry DMF, 1,4-phenylene dimethanol (3.2 g, 23.16 mmol) and Cs_2_CO_3_ (6.0 g, 18.41 mmol) were slowly added to the solution and reaction was stirred at 68 °C under nitrogen atmosphere for about 6 h and allowed to be cooled down to room temperature. Crude product was extracted with CHCl_3_/water several times and evaporated under vacuum. Residue was subjected to flash column chromatography (silica gel 40 g, EtOAc/Hexane 3:2) and 0.91 g of compound **3** was obtained as white solid. Then, 0.91 g of an alcoholic compound **3** was dissolved in 15 ml of freshly distilled dichloromethane and 0.14 ml of PBr_3_ was slowly added to the solution. The reaction was carried out at room temperature for about 2 h under nitrogen atmosphere. Crude product was extracted with 30 ml of water and dried under vacuum. White solid product **4** was successfully obtained in a quantitative yield without further purification for next step. KO*t*Bu (0.28 g, 2.49 mmol) and 11.2 ml of ethylene glycol were stirred at room temperature under nitrogen atmosphere. Then, 0.95 g of bromide compound **4** dissolved in 8 ml of dry THF was added slowly into the reaction mixture and the reaction was stirred at 60 °C for overnight. After cooling to room temperature, THF was evaporated and residue was extracted with CHCl_3_/water several times. Organic phase was evaporated under vacuum and residue was submitted to flash column chromatograpy (silica gel 40 g, EtOAc/Hexane 2:1) and 0.86 g of compound **5** was obtained as colorless oil in quantitative yield. Finally, to a mixture of 0.85 g of compound **5** and tosyl chloride (690 mg, 3.62 mmol) in 6 ml of dry dichloromethane, 0.64 ml of DIPEA and 4-(dimethylamino) pyridine (445 mg, 3.64 mmol) were added and reaction was carried out at room temperature for 2.5 h under nitrogen atmosphere. Dichloromethane was evaporated and crude product was directly subjected to flash column chromatograpy (silica gel 45 g, EtOAc/Hexane 2:1). 0.9 g of pure tosylate **6** (precursor of [^18^F]Flurpiridaz) was obtained by recrystallisation in dichloromethane at + 4 °C. Tosylate **6** was further purified through semipreparative HPLC for an accurate spectroscopic characterization (Fig. 1). Anal. Calcd for C_25_H_29_ClN_2_O_6_S: C, 57.63; H, 5.61; Cl, 6.80; N, 5.38; S, 6.15. Found: C, 57.86; H, 5.84; Cl, 7.03; N, 5.66; S, 6.34.

^1^H NMR ((CDCl_3_, 400 MHz) *δ* (ppm)): 7,80 (d, *J* = 9.1 Hz, 2H); 7,73 (s, 1H); 7,39 (d, *J* = 9.1 Hz, 2H); 5,29 (s, 2H,); 4,49 (s, 2H,); 4,20–4,19 (m, 2H); 3,70–3,65 (m, 2H); 2,42 (s, 3H); 1,60 (s, 9H).

#### Purification of precursor 6

**Fig. 1:**
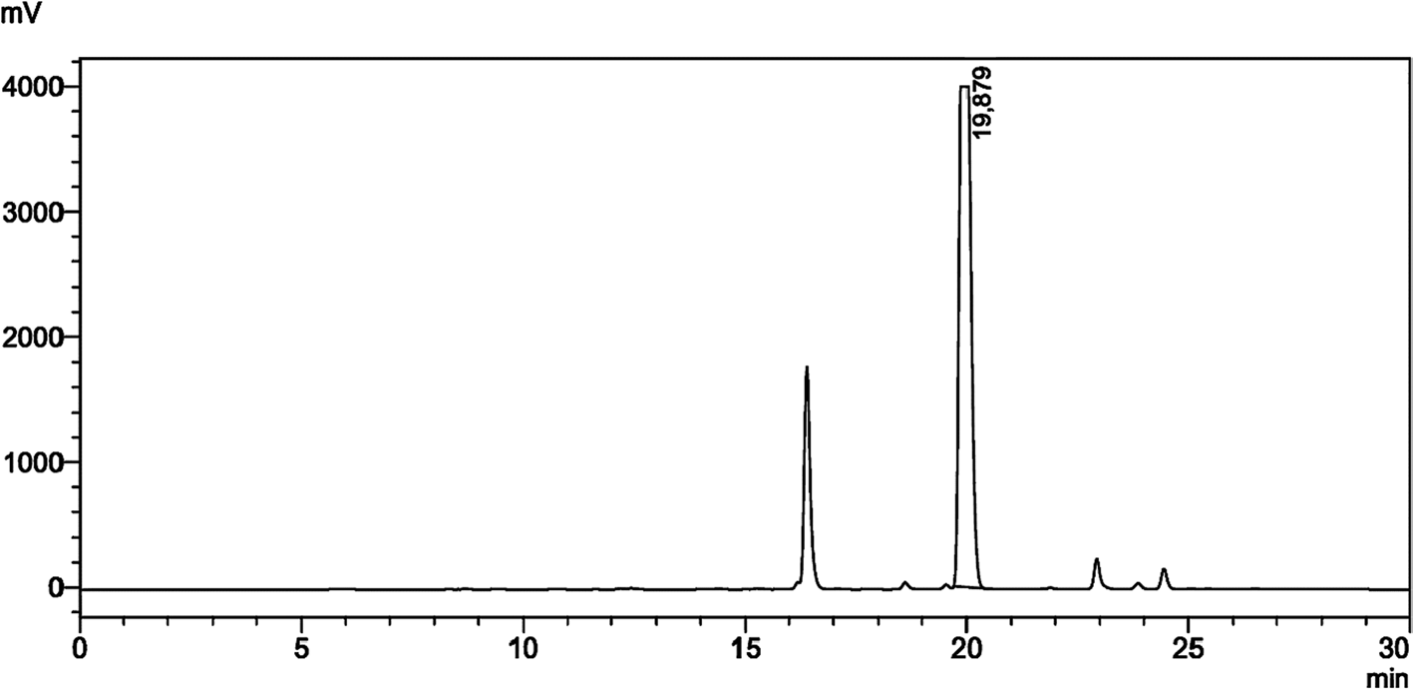
Purification (UV chromatogram) of precursor **6** (Method parameters: gradient flow; 0–10 min 40% B → 70% B, 10–20 min 70% B → 90% B, 20–25 min 90% B → 95% B, 25–30 min 95% B → 40% B (Phase A: H_2_O, Phase B: ACN), flow rate: 4 mL/min, UV: 320 nm, Column: GL Inertsustain C18 5 µm, 10 × 250 mm, Injection volume: 5 mL, Precursor **6** RT: ~ 20 min.)

### Characterization methods of precursor 6

#### HPLC analysis of precursor 6

**Fig. 2:**
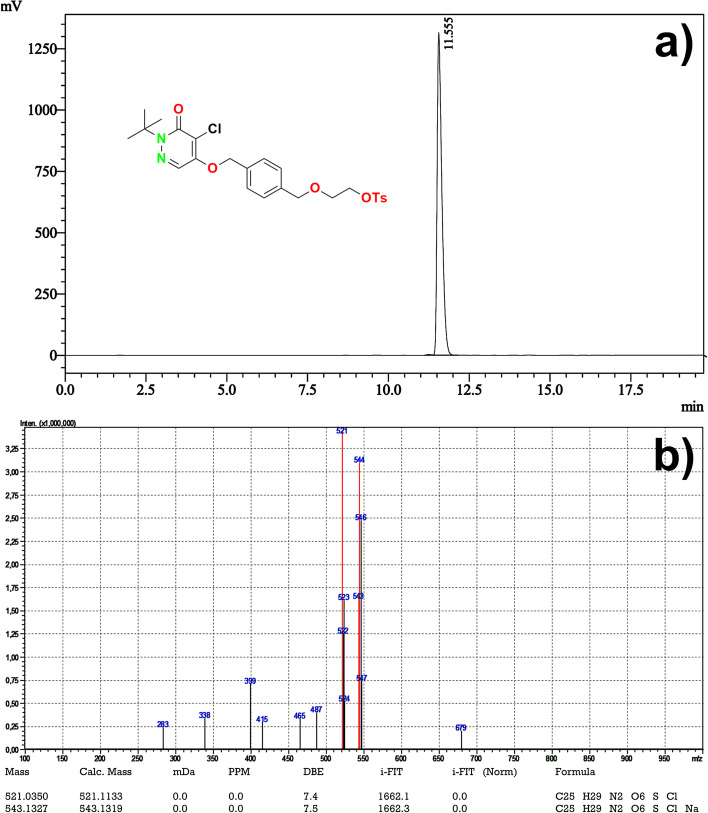
Chemical purity and identification (UV chromatogram) of precursor **6** (**a**), HR-MS spectrum of precursor **6** (**b**)

Chemical purity of the precursor was determined by HPLC analysis. Method parameters: Gradient flow: 0–15 min 40% B → 95% B,15–20 min 95% B (Phase A: H_2_O (0.1% TFA), Phase B: ACN (0.1% TFA). Flow rate: 0.6 mL/min, UV: 320 nm, Column: ACE C18 3 µm, 3 mm × 50 mm, Injection volume: 20 µL, Precursor **6** RT: 11.5 min (Fig. 2a).

#### HR-MS analysis of precursor 6

MS analysis was performed for precursor **6**. ESI–MS: calculated for C_25_H_29_ClN_2_O_6_S [M]:521.0350, found 521.1133, [M + Na] + 543.1327, found 543.1319 (Fig. 2b).

### Radiochemistry

#### Synthesis of [^18^F]Flurpiridaz (7)

##### Preliminary studies & synthesis of [^19^F]Flurpiridaz (7) (Cold runs)

*Materials* KF, Ethanol, and Acetonitrile were obtained from Sigma Aldrich. Kryptofix K2.2.2./K_2_CO_3_ (22 mg Kryptofix K2.2.2., 7 mg K_2_CO_3_, 300 µl acetonitrile and 300 µl pure water), TBA-HCO_3_ (0.075 M) solution and QMA Cartridges were from ABX. Sep-Pak C18 Plus Light Cartridge was from Waters.

#### Methods

Firstly, consecutive cold syntheses of [^19^F]Flurpiridaz **(7)** were performed using stable isotope fluorine-19 and optimum reaction parameters were tried to be determined**.**

#### Eluent solution-I (Kryptofix K2.2.2./K_2_CO_3_)

50 mg of KF was dissolved in 2 mL of ultrapure water and directly passed through the preconditioned QMA cartridge. The QMA cartridge was rinsed with 5 mL of ultrapure water and dried with N_2_. [^19^F]F trapped on the QMA cartridge was eluted into the reaction vial with 600 µL of Kryptofix K2.2.2./K_2_CO_3_ solution. Solvents in the reaction vial were removed at 100 °C, [^19^F]F and Kryptofix K2.2.2./K_2_CO_3_ were dried gently. Then, 10 mg of precursor **6** dissolved in 2 mL of anhydrous acetonitrile was added to the reaction vial and the mixture was sealed and heated at 95 °C for 10 min. The reaction solution was diluted with 5 ml of ultrapure water and directly passed through a preconditioned C-18 cartridge. C-18 cartridge was rinsed with 5 mL of ultrapure water and dried with air. Finally, C-18 cartridge was eluted with 5 mL of ethanol and transferred into the final product vial. The final product was diluted with 5 mL of ultrapure water (n = 3) and analyzed by HPLC (described in *HPLC analysis of precursor 6*) to determine its composition.

**Fig. 3:**
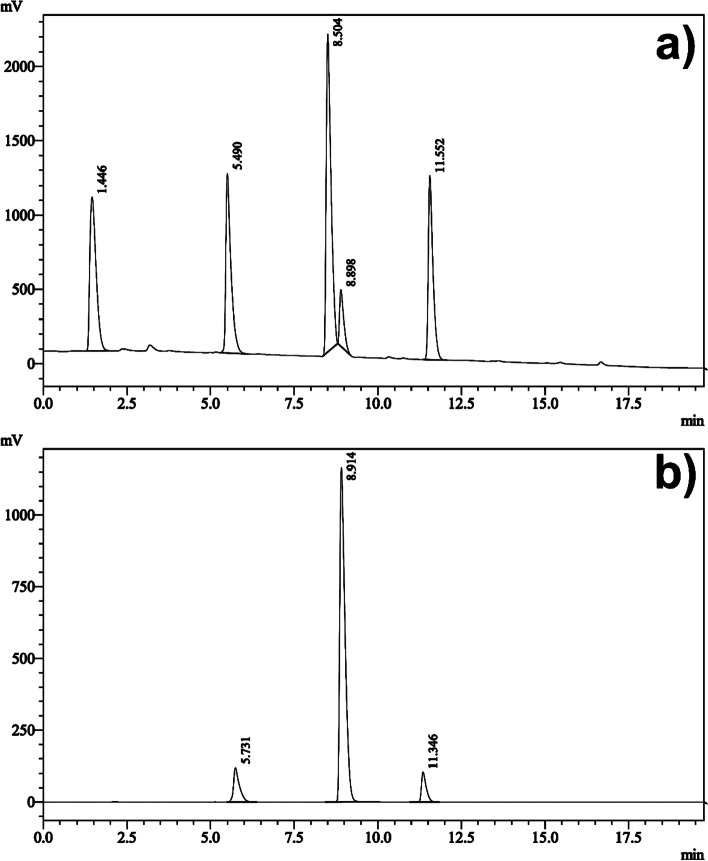
Final product compositions (UV Chromatograms), Fluoride elution method-I (Kryptofix K2.2.2./K_**2**_CO_**3**_) (**a**), Fluoride elution method-II (TBA-HCO_3_) (**b**)

Chromatogram analysis indicated that four different separate peaks were observed. The unreacted precursor **6** was detected around 11.55 min. The other two peaks were detected between 8 and 9 min. Another major peak around 5.5 min was detected. It was concluded that the chemical yield of product **7** was low due to the majority of side-product formations (Fig. 3a).

#### Eluent solution-II (TBA-HCO_3_)

50 mg KF was dissolved in 2 mL of ultrapure water and directly passed through the preconditioned QMA cartridge. The QMA cartridge was rinsed with 5 mL of ultrapure and dried with N_2_. [^19^F]F trapped on the QMA cartridge was eluted into the reaction vial with 750 µL of TBA-HCO_3_ solution (0.075 M). Solvents in the reaction vial were removed at 100 °C, [^19^F]F and TBA-HCO_3_ were dried gently. Then, 10 mg of precursor **6** dissolved in 2 mL of anhydrous acetonitrile was added to the reaction vial and the mixture was sealed and heated at 95 °C for 10 min. The reaction solution was diluted with 5 ml of ultrapure water and directly passed through a preconditioned C-18 cartridge. C-18 cartridge was rinsed with 5 mL of ultrapure water and dried with air. Finally, the C-18 cartridge was eluted with 5 mL of ethanol into the final product vial. The final product 7 was diluted with 5 mL of ultrapure water (n = 3) and analyzed by HPLC (described in *HPLC analysis of precursor 6*) to determine its composition.

A clear major peak around 9 min was detected which belongs to [^19^F]Flurpiridaz (**7**). The unreacted precursor **6** was detected around 11.40 min. A possible side-product was detected around 5.5 min. as in the elution method-I, but there was relatively a small amount in the final product composition. Those analyses proved that the chemical yield is high, and the amount of side-product formation is quite low (Fig. 3b).

**Fig. 4:**
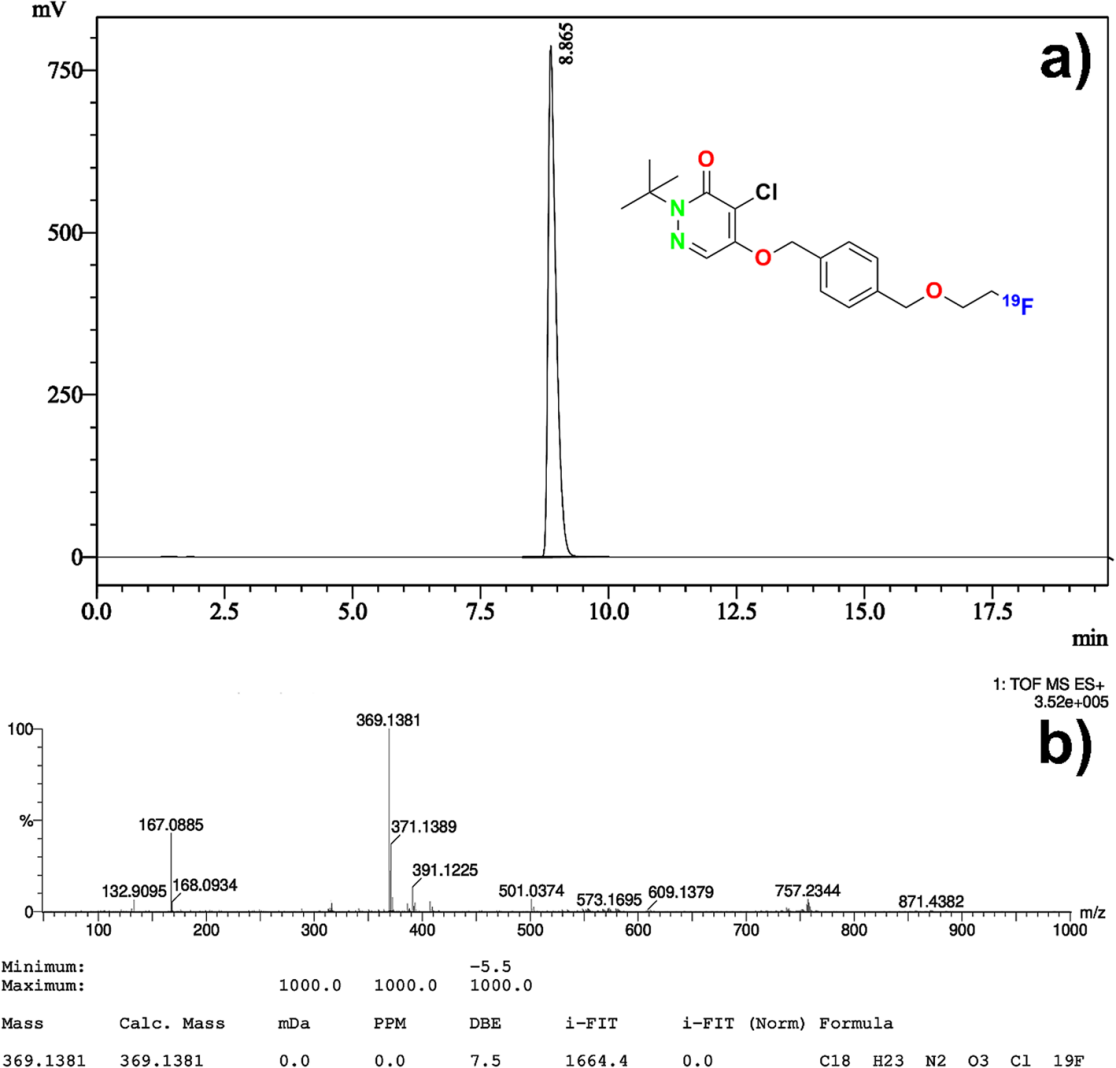
HPLC UV chromatogram and identification of [^19^F]Flurpiridaz (**7**) (**a**), HR-MS analysis of [^19^F]Flurpiridaz (**7**) (**b**)

In this study, the substance obtained around the 9 min. was purified and isolated from the final product composition (Fig. 3b). HR-MS analysis was performed for the isolated concentrated pure substance (Fig. 4a) and it was clearly identified as [^19^F]Flurpiridaz (**7**) (Fig. 4b). The second method was chosen as the elution method for the synthesis of [^18^F]Flurpiridaz (**7**).

##### Automated synthesis procedure of [^18^F]Flurpiridaz (7)

*Materials* Ethanol, *L*-ascorbic acid, and Acetonitrile were obtained from Sigma Aldrich. WFI, TBA-HCO_3_-Solution, and QMA Cartridges were from ABX. O-18 Water was purchased from CMR. Disposable consumables, cassettes, and other accessories were obtained from Eckert & Ziegler. Other Sep-Pak Cartridges, tC18 Plus Long Cartridge, Sep-Pak Alumina N Plus Light Cartridge were from Waters.

#### Method

We have developed an automated radiosynthesis procedure for [^18^F]Flurpiridaz (**7**) on the ML PT synthesis device. Each step has been optimized with repetitive studies. The final product was isolated as a pure and sole product without further preparative HPLC purification process. Detailed synthesis steps such as [^18^F]F trapping, [^18^F]F elution, [^18^F]F drying, radiolabeling, purification and product transfer were well described in Table 1.

**Table 1:** Radiosynthesis steps of [^18^F]Flurpiridaz (**7**)

Step		Reagents	Parameters
1	Trapping of [^18^F]F on the QMA cartridge	QMA cartridge, O-18 water	The irradiated O-18 water is vacuumed over the QMA cartridge into the O-18 recovery vial for 90 s
2	Drying of the QMA cartridge	QMA cartridge, N_2_	The QMA cartridge is dried with high flow N_2_ for 90 s
3	Elution of [^18^F]F to the reaction vessel	750 µL of TBA-HCO_3_-Solution (0.075 M)	
4	Evaporation of solvents and drying process		Solvents are evaporated under vacuum and low flow N_2_ for 6 min
5	Transfer of precursor to the reaction vessel	10 mg of precursor in 2 mL of anhydrous ACN	
6	Radiolabeling		The reaction vessel is sealed and heated at 95 °C for 10 min
7	Loading of the reaction mixture into the purification cartridge tC18	tC18 cartridge (pre-conditioned with 10 mL of ethanol, rinsed with ultrapure water, and dried with air) WFI	The reaction mixture is diluted with 6 mL of WFI and passed through tC18 The reaction vessel was rinsed a second time with 5 mL of WFI and passed through tC18 Finally, tC18 is rinsed with 10 mL of WFI and dried with N_2_ [^18^F]Flurpiridaz, unlabeled precursor, and side products are trapped on the tC18 cartridge
8	Rinsing of tC18 cartridge with purification solution	16 mL (40% ethanol/60% WFI, 50 mg/mL of Ascorbic Acid)	The more polar side-product is washed into the waste vial from tC18 Flow rate: 1 mL/min 20 min
9	Elution of [^18^F]Flurpiridaz into the 30 mL syringe from tC18	5 mL (50% ethanol-50% WFI)	[^18^F]Flurpiridaz is eluted into the 30 mL syringe Flow rate: 2 mL/min Almost entire of the more apolar unlabeled precursor remains in the tC18 cartridge, while [^18^F]Flurpiridaz is eluted by polarity difference
10	Dilution of [^18^F]Flurpiridaz in the 30 mL syringe	20 mL of WFI	The precursor has no water solubility. The final product is diluted with 20 mL of WFI. In this way, Even a trace amount of precursor is precipitated
11	Transfer of the final product	The final vial content, 5 mLof WFI (1500 mg of Ascorbic Acid buffer pH: 6.7) The final purification cartridge: Sep-Pak Alumina N Plus Light Cartridge Two pieces of 0.2-micron sterilization filter	25 mL of the final product [^18^F]Flurpiridaz is transferred to the final product vial, passing through the final purification cartridge and two sterilization filters Free [^18^F]F is removed by Alumina N cartridge. The trace amount of precursor precipitated is removed by two pieces of sterilization filter

**Table 2:** Description of the schematic synthesis diagram (Fig. 5)

1	Low Flow N_2_ (18–40 mL/min)	12	Chemical valve (open/close)
2	High Flow N_2_ (300–500 mL/min)	13	Vacuum sensor
3	750 µL of TBA-HCO_3_-Solution (0.075 M)	14	Reactor, Heater
4	O-18/F-18 V-Vial	15	O-18 Recovery Vial (10 mL)
5	Sep-Pak Light QMA Cartridge	16	10 mg of precursor in 2 mL of anhydrous ACN
6	250 mL of WFI	17	Waste Vial
7	5 mL (50% Ethanol—50% WFI)	18	16 mL (40% ethanol / 60% WFI, 50 mg/mL of Ascorbic Acid)
8	Sep-Pak tC18 Plus Long Cartridge	19	Sep-Pak Alumina N Plus Light Cartridge
9	Radioactivity Detector	20	0.2 Micron Sterilization Filters
10	Vacuum Pump	21	Final Product Vial (30 mL) + 5 mL 1500 mg Ascorbic Acid Buffer
11	Waste Bottle (250 mL)	22	10 mL Syringe

### Quality control of [^18^F]Flurpiridaz (7)

Radiochemical and chemical purity analyses of the final product [^18^F]Flurpiridaz **(7)** were performed by Radio-HPLC (described in *HPLC analysis of precursor 6*) (Fig. 6). In addition, after the synthesis, the composition of the waste vial was analyzed by Radio-HPLC. it was determined that free [^18^F]F and other [^18^F]F side products were clearly removed (Fig. 7).

**Fig. 5:**
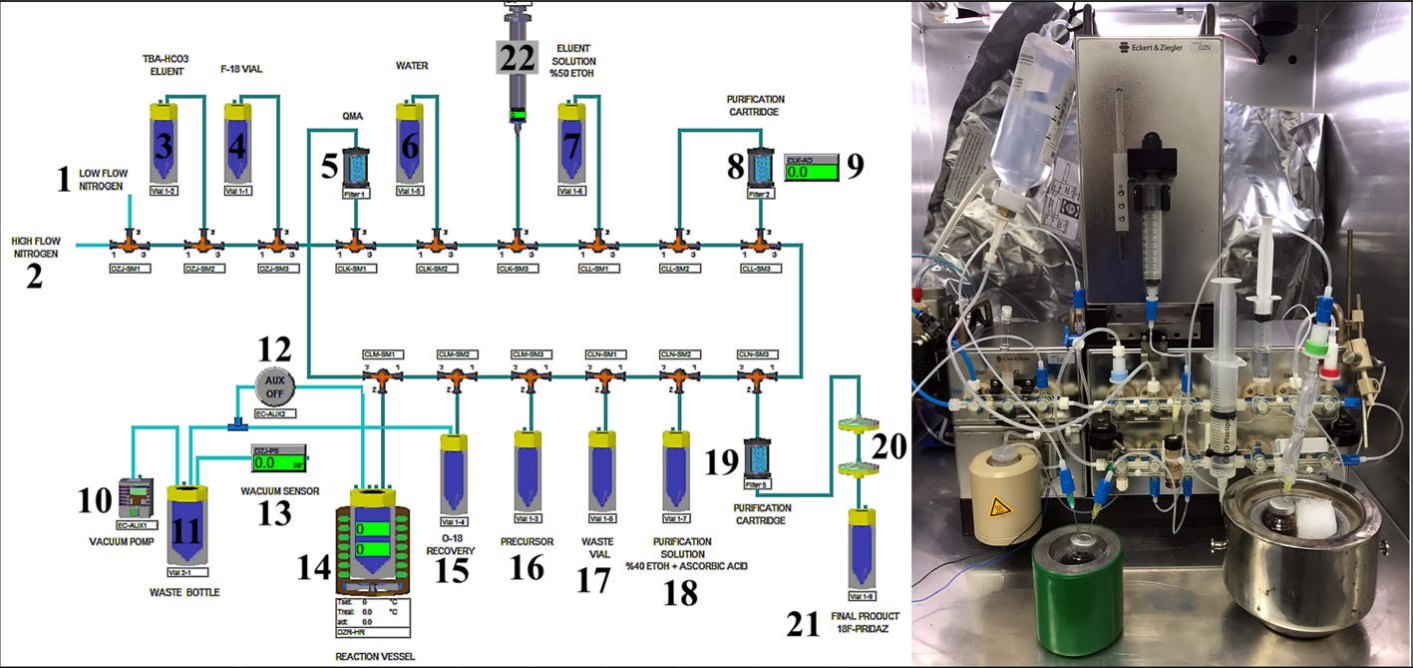
Schematic HMI diagram of the automated synthesis of [^18^F]Flurpiridaz (**7**). And the photo of the cassette, reagents, and ML PT synthesizer

**Fig. 6:**
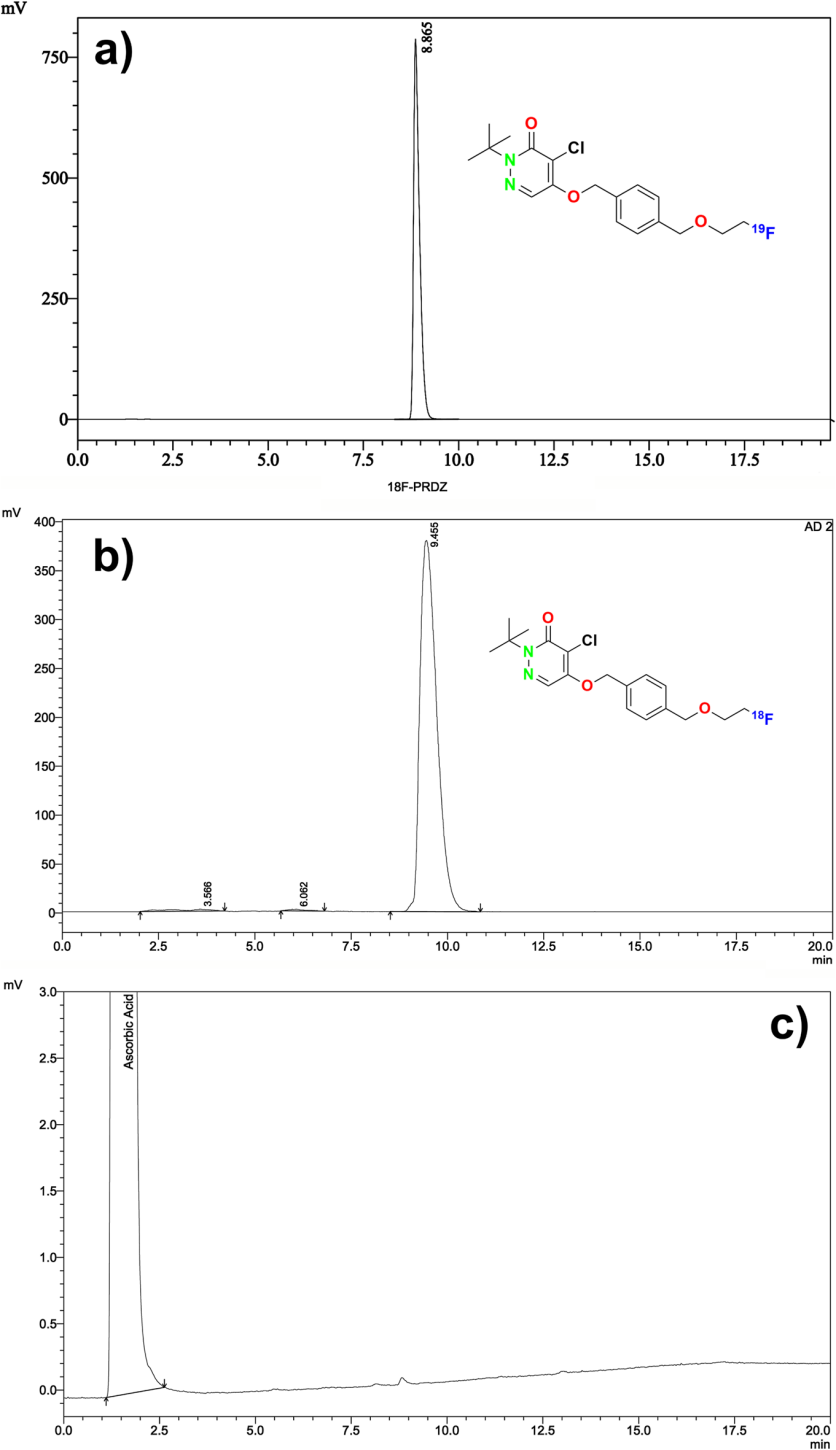
Radio-HPLC chromatogram and identification of [^18^F]Flurpiridaz (**7**) (**b**), HPLC–UV chromatogram of [^18^F]Flurpiridaz (**7**) (**c**), HPLC–UV chromatogram of [^19^F]Flurpiridaz (**a**) (**7**)

**Fig. 7:**
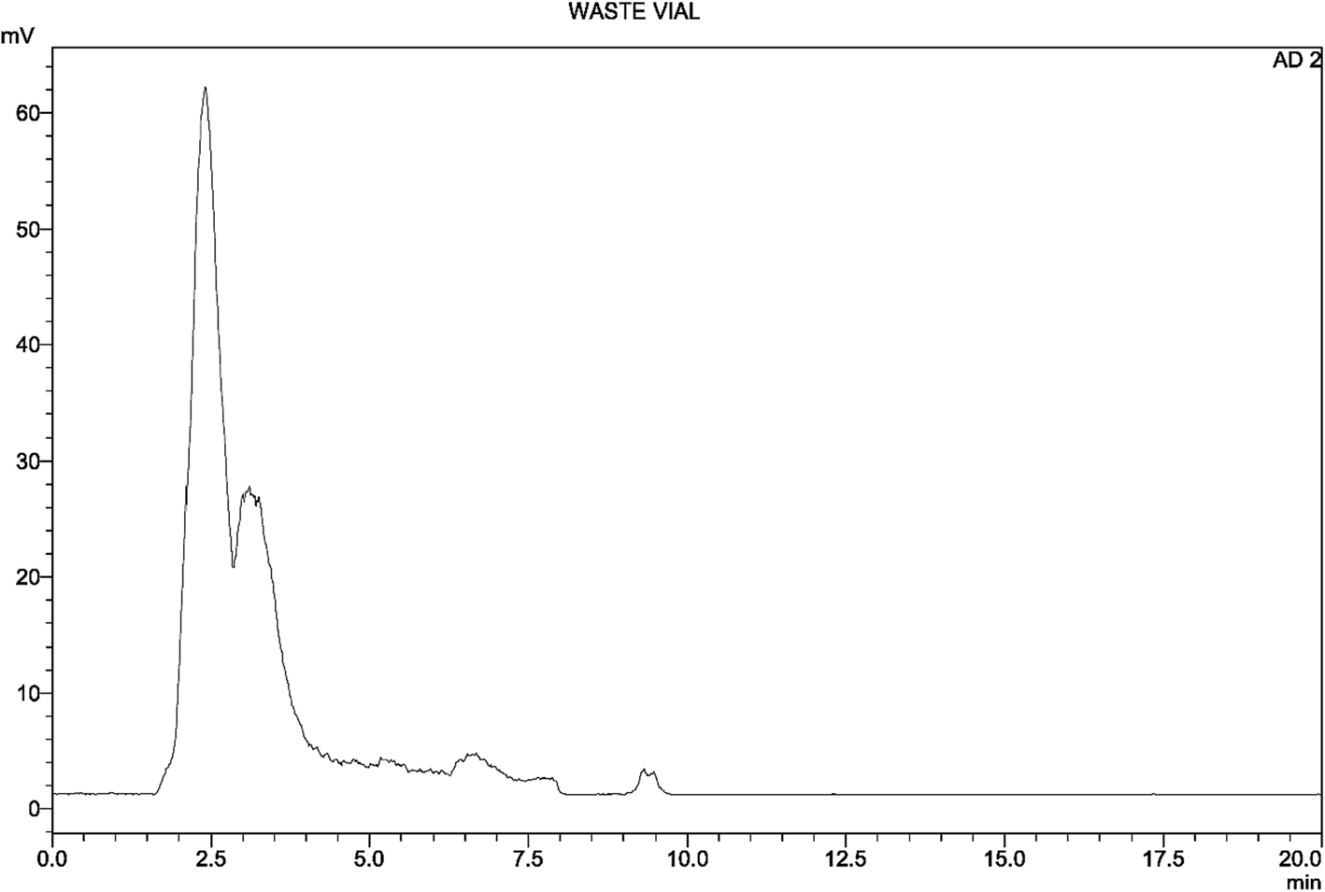
Radio-HPLC chromatogram of the waste vial

## Results

Organic synthesis of precursor **6** was achieved in our laboratory with more than 99% chemical purity and a total yield of 35%. Structural analysis was confirmed by NMR, elemental analysis, and HR-MS which was consistent with previously published literature (Ahmed et al. 2020). Manually cold labeling experiments were afforded before the synthesis of [^18^F]Flurpiridaz (**7**) starting from K^19^F for optimization. HPLC UV and HR-MS results revealed that the nature of PTC was a dramatic effect on optimization and TBA-HCO_3_ PTC increased the fluorination yield with a minimum amount of side product (Fig. 3b)**.** Parameters obtained by cold runs were tried to adapt ML PT synthesis module for hot runs (Tables 1, 2, 3). A fully automated radiosynthesis of [^18^F]Flurpiridaz (**7**) was validated by three sequential syntheses. Table 3 and Fig. 5a presented that RCY was nearly 65% with more than 98% RCP. Table 4 summarizes the specifications of desired product **7** and entire results such as residual solvent, sterility, RCP, and BET are consistent with general radiopharmaceutical quality guidelines.

**Table 3:** General synthesis parameters (n = 3)

Total duration of the synthesis	110 min
Radiolabeling yield (Decay corrected)	55–65%
Radiolabeling yield (Uncorrected)	25–35%
Final product volume	30 ml

**Table 4:** Final product specifications of [^18^F]Flurpiridaz (**7**)

Test	Method	Acceptance criteria	Average results
Appearance	Visual	Clear, Colorless, or Light Yellow	Clear, Colorless, or Light Yellow
pH	pH-Indicator Strip	4.5–8.5	6–7
Half-Life	Half-Life Measurement	105–115 min	109 min
Radiochemical Purity	Radio-HPLC	> 95%	98.5%
Radionuclidic Purity	Gamma Spec	> 99.9%	> 99.9%
Precursor	HPLC	N.D	N.D
(TBA)	Spot Test	≤ 2.6 mg/V	≤ 2.6 mg/V
Ethanol	GC	≤ 10% (h/h)	8.2% (h/h)
Acetonitrile	GC	≤ 4.1 mg / V	0.04 mg / V
Ascorbic Acid	HPLC	≤ 500 mg / V	151 mg / V
BET	LAL	< 175 IU/V	< 175 IU/V
Sterility	Sterility Test	Sterile	Sterile

*V: 3 mL

### Stability studies of [^18^F]Flurpiridaz (7)

**Fig. 8:**
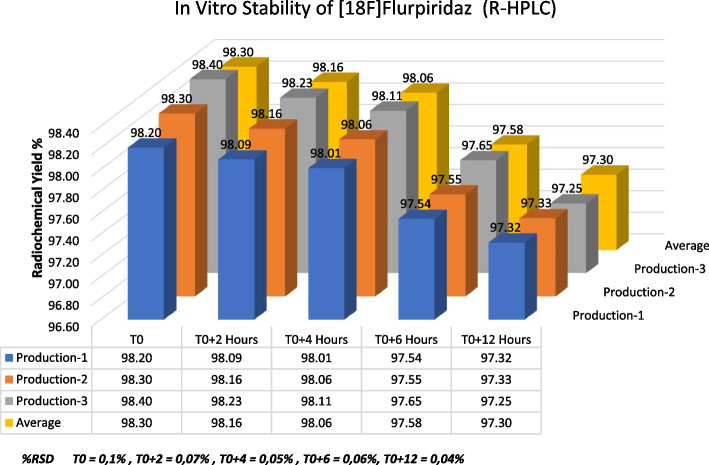
Stability studies of [^18^F]Flurpiridaz (**7**) (T0 = End of the synthesis, n = 3)

After a successful validation of the synthesis of fluorinated product **7**, stability experiments were exhibited. Radiochemical purity analyzes were performed by Radio-HPLC at 2-h intervals from the end of the synthesizes (T0) for three separate productions. The final product [18F]Flurpiridaz (7) was observed for 12 h within the frame of this plan in room conditions. [^18^F]Flurpiridaz (**7**) remained within radiopharmaceutical quality during this time. The entire analysis results, the average of the analysis results, and the RSD% are depicted in Fig. 8.

## Discussion

The radiolabeling procedure of radiopharmaceutical preparation is **a** critical case before clinical administration (De Decker and Turner 2011). Currently, majority of the radiopharmaceuticals are not commercially available and their “*in house preparation*” was mainly based on manual synthesis although manually process causes radiation exposure and contamination risk (Meyer et al. 2004). On the other hand, a fully automated preparation of radiopharmaceuticals provides standardization, minimal radiation exposure, validation, reproducibility, and high yield (Velikyan et al. 2015). Furthermore, a fully automated system facilitates a GMP-compliant production in clinical studies and disposable cassette systems are employed to prevent cross-contamination caused by tubing systems, which leads to sterile and highly pure radiolabeled compound. (Boschi et al. 2013).

Modular Lab-PharmTracer (ML PT) synthesizer is a fully user-defined system combined by activity detectors, valves, pressure sensor, pump, SPE cartridge and heater. All systems are controlled by an electric cabinet tool with an easy software program namely Modular-Lab. An appropriate system configuration enhances an efficient option for preparation of popular fluorine-18 based tracers such as [^18^F]F-FES, [^18^F]F-FDG, [^18^F]F-PSMA-1007 and[^18^F]F-FLT.

**Scheme 2:**
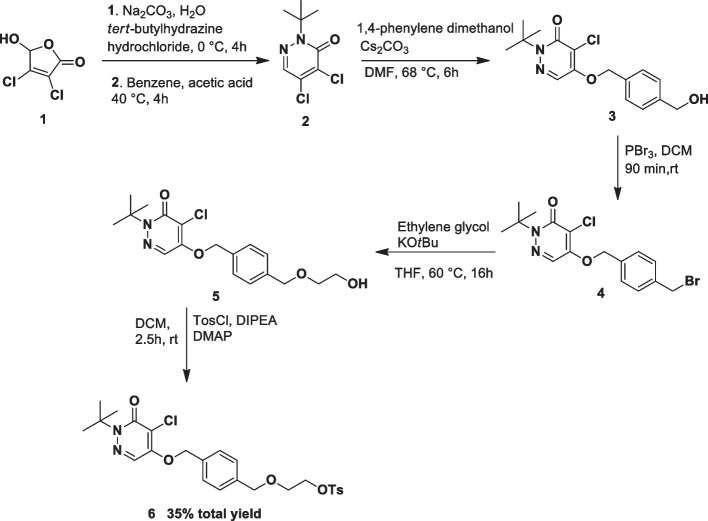
Synthesis of precursor (**6**) of [^18^F]Flurpiridaz starting from mucochloric acid (**1**)

In light of the routine an automatically production of above fluorine-based compounds, we aimed to develop a fully automated synthesis of [^18^F]Flurpiridaz **(7).** Firstly, stepwise organic synthesis of flurpiridaz precursor (**6**) was achieved with a total yield of 35% and confirmed by NMR and HR-MS (Scheme 2).

After pure isolation of precursor **6**, optimized condition was aimed for radiolabeling of precursor **6**. In this work, amount of precursor, solvent type, reaction time and temperature were kept constant due to the previously published comprehensive study (Cesati et al. 2015). Firstly, cold experiments were tried to optimize reaction conditions without radioactive exposure. Stable isotope [^19^F]F was employed for nucleophilic substitution reaction. ^19^Flouride was trapped with QMA cartridge followed by elution with Kryptofix K2.2.2./K_2_CO_3_ as a phase transfer catalyst (PTC) which is widely used in [^18^F]flourine radiolabeling reactions. HPLC UV analysis (Fig. 3a) indicated that there was a dramatic increase of unexpected side products and a low amount of fluorination yield was observed. On the contrary, the chemical yield of fluorination increased with a minimum amount of side products in the presence of TBA-HCO_3_ (Fig. 3b). The main reason for this result is that HCO_3_^−^ decreased the hydrolysis rate and increased the fluorination yield (Fig. 3a). Unfortunately, the basicity of K_2_CO_3_ caused the decomposition of the product and reduced fluorination yield. (Vavere et al. 2018). HR-MS and HPLC spectra identified relative quantity taking part in a reaction and precursor **6**, side products, and [^19^F]Flurpiridaz **(7)** were well determined (Fig. 3,4).

**Scheme 3:**
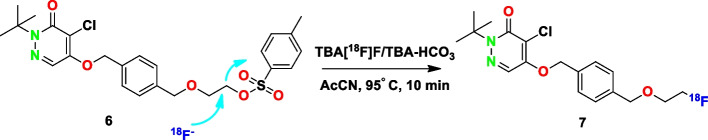
S_N_2 reaction of precursor **6**

After a sequential manually cold synthesis of [^19^F]Flurpiridaz (**7**), an automated synthesis of radioactive fluorine compound was tried and each step was optimized with consecutive studies (Table 1). Fluorination of the precursor **6** is formed by S_N_2 nucleophilic substitution reaction. The nucleophile [^18^F]flouride attacks the backside of the carbon atom and the tosylate group was easily separated from the electrophilic carbon atom (Scheme 3). In general, use of polar aprotic solvents such as acetonitrile is very important for the activity of nucleophiles in S_N_2 nucleophilic substitution reactions. Polar protic molecules such as water and ethanol make hydrogen bonds with nucleophiles, and they reduce the activity of nucleophiles and the yield of the labeling reaction. The cleavage of hydrogen bonds has to be afforded so that the nucleophile can attack to the electrophilic carbon. The fluoride ion, a weak nucleophile, is dramatically affected by the presence of impurities even in trace amounts during the labeling process and the reaction yield decreases. Therefore, evaporation of solvents and drying of TBA-[^18^F]F & TBA-HCO_3_ after QMA cartridge elution is a very critical process for desirable radiolabeling yield. The nature of the solvent is another critical parameter and anhydrous acetonitrile has to be used for high radiochemical yield.

Carefully spectral analyses showed that there were four main components in the post-reaction mixture; free [^18^F]F, [^18^F]Flurpiridaz **(7)**, non-radiolabeled precursor **6**, and one side-product (Fig. 3** (b)**). In general, combined preparative HPLC system is frequently utilized for the purification of the final fluorinated product in literature (Wang et al. 2020). Even this purification system has significant advantage for purification, this system is quite expensive, and a larger hot cell is required. For this reason, we aimed to design a simplified system for fast purification without using preparative HPLC. In our purification method, after the completion of radiolabeling, the reaction mixture was loaded onto the tC18 cartridge, and free fluorine and other polar impurities were transferred into the waste vial by water. Impurities which were slightly more polar than [^18^F]Flurpiridaz (**7**) were washed into the waste vial by 16 ml of purification solution (40% ethanol/60% WFI, 50 mg/mL of ascorbic acid). At this step, the flow rate and ethanol concentration are another critical case for the exact separation of substances. The ascorbic acid in the solution was used to prevent radiolysis on the tC18 cartridge during this process. Then, the final product [^18^F]Flurpiridaz (**7**) was eluted with 5 ml of solution (50% ethanol-50% WFI) into a 30 ml capacity injector with a certain flow rate. In this step, slightly non-polar precursor **6** also remained on the tC18 cartridge. Precursor **6** is not water-soluble, for this reason, an additional 20 ml of water was sent to the injector to precipitate precursor **6**, in case even trace amounts of precursor **6** are eluted with the final product. Finally, [^18^F]Flurpiridaz (**7**) was successfully transferred to the final product vial through the alumina cartridge and two 0.2-micron sterilization filters. The alumina cartridge removed free [^18^F]F residue, while the 0.2-micron filters prevented the precipitated precursor **6** from passing into the final product vial. The final product consists of [^18^F]Flurpiridaz (**7**), 7–8% ethanol, and 50 mg/ml of ascorbic acid with a total volume of 30 ml. Ethanol and ascorbic acid are important for final product stabilization. [^18^F]Flurpiridaz (**7**) was produced with decay-corrected 55–65% radiochemical yield, > 98% radiochemical purity, and in radiopharmaceutical quality (Tables 3, 4). Stability studies have demonstrated that the final product protected its stability with initial radiopharmaceutical quality for 12 h (Fig. 7).

## Conclusion

In conclusion, a fully automated synthesis of [^18^F]Flurpiridaz (**7**) without further preparative HPLC purification on ML PT has been successfully described for the first time. Highly pure precursor **6** was synthesized with a total yield of 35% and well characterized by NMR and HR-MS. The carefully sequential set of cold trials were performed for optimization conditions before the hot reactions, and TBA-HCO_3_ increased chemical yield with a minimum amount of side products. The evaluation of hot reaction records has proved that our automated synthesis method without further preparative HPLC purification enables the production of [18F]Flurpiridaz with > 98% radiochemical purity. All synthesis and purification steps were achieved without any manual interaction. Disposable cassette was employed to provide a limit of dose standard. The desirable radiopharmaceutical quality of the product was obtained without using a preparative HPLC system. Stability experiments were well exhibited and final specifications were recorded according to the acceptance criteria in Ph. Eur. regulations. This simplified system provided cost effectiveness and could lead to a practical diagnostic application for multicenter clinical trials.

## Data Availability

All data regarding characterization and analyses during this work are included in this published article.

## Abbreviations

ACN: Acetonitrile
CAD: Coronary artery disease
CMR: Cardiac magnetic resonance
DCP: 2-(Tert-butyl)-4 5-Dichloropyridazin-3(2 h)-one
DCM: Dichloromethane
DIPEA: *N*,*N*-Diisopropylethylamine
DMF: Dimethyl Formamide
EtOAc: Ethyl acetate
HPLC: High performance liquid chromatography
HR MS: High resolution mass spectrometry
KF: Potassium fluoride
MC: Mitochondrial complex
ML PT: Modular Lab-PharmTracer
MPI: Myocardial perfusion imaging
NMR: Nuclear magnetic resonance
PET: Positron emission tomography
Ph Eur: European pharmacopeia
QC: Quality control
QMA: Quaternary methyl ammonium
SPECT: Single-photon emission computed tomography
TBA-HCO_3_: Tetrabutylammonium bicarbonate
TFA: Trifluoro acetic acid
THF: Tetrahydrofuran
TLC: Thin-layer chromatography
UV: Ultra violet
WFI: Water for injection
